# Second Child After Pregnancy and Lactation-Related Osteoporosis (PLO) Treated With Teriparatide: A Case Report

**DOI:** 10.7759/cureus.64900

**Published:** 2024-07-19

**Authors:** Shinsuke Yamada, Noriyuki Hayashi, Norikazu Toi, Daichi Miyaoka, Motomu Hashimoto

**Affiliations:** 1 Clinical Immunology, Osaka Metropolitan University of Medicine, Osaka, JPN; 2 Internal Medicine, Ohno Memorial Hospital, Osaka, JPN; 3 Metabolism, Endocrinology, and Molecular Medicine, Osaka Metropolitan University of Medicine, Osaka, JPN; 4 Immunology and Genomics, Osaka Metropolitan University of Medicine, Osaka, JPN

**Keywords:** second pregnancy, teriparatide, aggressive treatment, bone mineral density, pregnancy and lactation-related osteoporosis

## Abstract

Pregnancy and lactation-associated osteoporosis (PLO) is one of the rarest and most serious primary osteoporotic conditions that develops during the third trimester of gestation and/or early postpartum period. A history of PLO often causes individuals to hesitate when considering an additional child due to anxiety about recurrence risk. Reported here are details of a 29-year-old patient with PLO given aggressive treatment for osteoporosis with teriparatide who subsequently gave birth to a healthy child at age 33 without PLO recurrence. Further case studies are needed to examine the efficacy and safety of PLO treatment and to provide beneficial results for those who wish to have another pregnancy.

## Introduction

Pregnancy and lactation-related osteoporosis (PLO), one of the primary osteoporosis conditions that develops during the third trimester of gestation and/or early postpartum period, is extremely rare, with an estimated incidence of about 0.4 per 100,000 pregnant women [1]. Although serious, because of associated compression fractures of the vertebrae, PLO responds well to osteoporosis drugs [2-6], and affected patients generally have a very good long-term prognosis. On the other hand, excruciating back and lower back pain that follows the onset of the disease can make postpartum childcare difficult, and patients may need to focus on caring for themselves when the symptoms are severe. Because of such experiences, PLO patients and their families tend to hesitate when considering another pregnancy.

Reported here are details of a patient who developed PLO after giving birth to her first child and then successfully gave birth to a second child without recurrence following aggressive osteoporosis treatment with teriparatide. Although several studies have noted the therapeutic effects of osteoporosis drugs for PLO patients, there are few reports of new pregnancies and births following the onset of the condition.

## Case presentation

**Figure 1:**
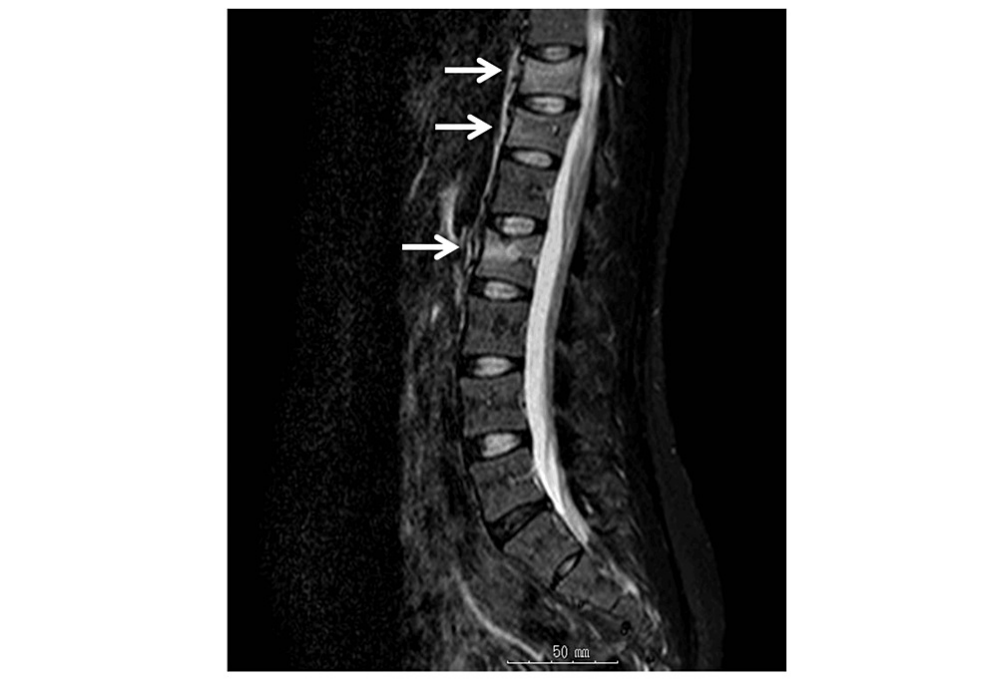
Magnetic resonance imaging (MRI) findings at pregnancy and lactation-associated osteoporosis (PLO) diagnosis. Multiple compression fractures of the 11th and 12th thoracic vertebrae, and second lumbar vertebrae were noted.

A healthy Japanese woman with no specific medical or family history experienced her first pregnancy at the age of 29 and gave birth to a child by normal delivery. Approximately one month following delivery, she became aware of uncontrolled lower back pain, which led to walking difficulties approximately two months postpartum. Magnetic resonance imaging (MRI) of the thoracolumbar spine performed at a local hospital revealed multiple compression fractures of the 11th and 12th thoracic vertebrae and the second lumbar vertebrae (Figure 1).

Further test findings were negative for malignant, hematologic, collagen/rheumatic, or endocrine/metabolic diseases. However, a dual-energy x-ray absorptiometry (DXA) examination showed a marked decrease in bone mineral density (BMD) in the lumbar spine (young adult mean (YAM) = 70%) and femoral neck (YAM = 68%). The YAM values are the average BMD values (reference values) for young adults at 100% and are expressed as a percentage compared to the subject BMD values. The patient was subsequently diagnosed with PLO and referred to our hospital on postpartum day 160. Until that time, there had been no therapeutic intervention performed for osteoporosis, and acetaminophen alone was being administered for pain management.

An MRI scan of the thoracolumbar spine performed at the first visit to our hospital revealed a new compression fracture of the eighth thoracic vertebra. The child was immediately weaned, and the patient was treated with eldecalcitol (0.75 ug/day) from postpartum day 161. However, a DXA test repeated 193 days postpartum showed a further decrease in lumbar spine BMD (0.639 g/cm^2^, YAM = 63%) and femoral neck BMD (0.395 g/cm^2^, YAM = 67%). The patient stated a desire to have a second baby in the future; thus, eldecalcitol was discontinued and daily teriparatide (20 ug/day) was started from postpartum day 203 to strengthen the treatment regimen for osteoporosis. Six months after beginning teriparatide treatment (13 months postpartum), DXA showed a marked increase in both lumbar spine (YAM = 77%) and femoral neck (YAM = 78%) BMD. In addition to drug therapy, she was careful to consume foods containing calcium (Ca) and vitamin D and did walking exercise with approximately 8000 steps most days. The daily teriparatide regimen was completed after 24 months (31 months postpartum), and a DXA examination showed further improvements in BMD in both the lumbar spine (YAM = 84%) and femoral neck (YAM = 82%).

**Figure 2:**
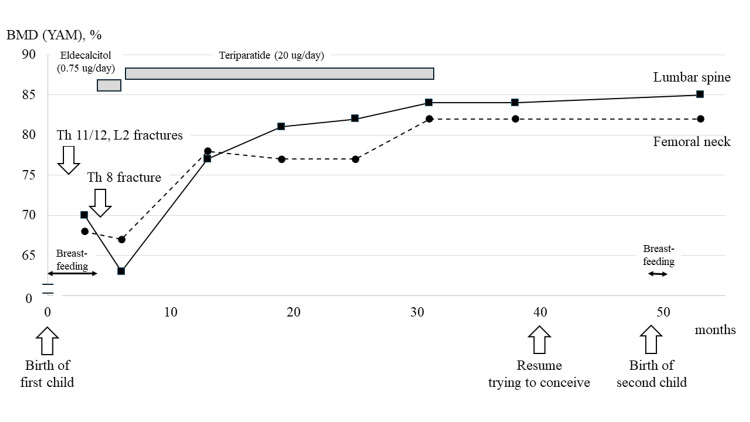
Clinical course of pregnancy and lactation-associated osteoporosis (PLO) treatment and bone mineral density (BMD). Following treatment intervention with teriparatide, no new compression fractures occurred. Furthermore, bone mineral density (BMD) was markedly increased in both the lumbar spine and femur neck, which was maintained without any decreases noted following the birth of the second child. YAM: young adult mean

The patient maintained her desire to have a second child and was followed without alternative maintenance therapy after ending teriparatide treatment. Seven months after the end of teriparatide treatment (38 months postpartum), lumbar spine (YAM = 84%) and femoral neck (YAM = 82%) BMD were found to be well maintained. At 40 months after the birth of the first child, pregnancy with a second child was confirmed, and nine months later (49 months postpartum), a healthy baby was delivered in a normal manner. Breastfeeding was performed for only one month. A DXA examination at four months after delivery of the second child showed no decrease in bone density, with YAM values for BMD in the lumbar spine and femoral neck of 85% (BMD = 0.858 g/cm^2^) and 82% (BMD = 0.645 g/cm^2^), respectively, thus follow-up examinations for PLO were completed. A summary of this case is presented in Figure 2.

## Discussion

PLO is a very rare type of osteoporosis, first reported by Nordin et al. in 1955 [7]. It occurs in young women during the third trimester of gestation and/or the early postpartum period and is classified as a primary osteoporosis condition associated with postmenopausal osteoporosis. A family history of PLO, smoking habit, low body weight, and lack of Ca intake have been identified as risk factors, though the etiology remains largely unknown, and even pregnant women with no known risk factors for osteoporosis can develop the disease without a specific known trigger. The present patient had no smoking or drinking habit, no history of fractures, and no family history of PLO. She had a slender appearance (height 154 cm, weight 49 kg, body mass index (BMI) 20.7 kg/m2), though she was naturally healthy with no history of taking warfarin or steroids, nor complications considered to possibly lead to secondary osteoporosis. There was a history of physical activity during her school years, and she enjoyed a variety of types of food without excessive caffeine intake or dieting. While loss of Ca during pregnancy and lactation in women with low bone mass may trigger the onset of PLO, BMD had never been determined before onset, so that factor is unknown in this case.

Bones during pregnancy are protected by increased secretion of the female hormones estrogen and progesterone from the placenta, as well as increased production of 1.25 (OH) 2D due to activation of 1α hydroxylase by placental hormones [8]. Fetal Ca demand is markedly increased in late pregnancy; thus, when the transfer of Ca from the maternal system cannot match the demand, secondary hyperparathyroidism is induced [9,10] and maternal bone density decreases [11,12]. After birth, in addition to the loss of the placenta, maternal Ca absorption from bone is further enhanced by parathyroid hormone-related peptide (PTH-rp), which is produced in mammary tissue by suckling stimulation during lactation [13]. Maternal Ca loss related to lactation has been estimated to range from 200-300 mg/day [14], with lactation over a period of six months resulting in a 3-6% decrease in BMD [11]. These factors can cause a substantial amount of BMD loss in postpartum women within a short period of time as compared to postmenopausal women, who have a loss rate of 1-2% per year, though with weaning and recovery of ovarian function, BMD improves at a rapid rate to its pre-pregnancy state [15].

The present patient had no placental function abnormalities, while breastfeeding was not excessive at approximately 10 feedings per day and menstruation had resumed. However, BMD continued to decline even at six months postpartum; thus, an adequate spontaneous recovery in the future was considered unlikely, and aggressive treatment for osteoporosis was started. Although there is no established drug therapy for PLO, nearly all medications given for osteoporosis, such as active vitamin D [2], bisphosphonate preparations [3], teriparatide [4], denosumab [5], and romosozumab [6], have been reported to be safe and show good therapeutic efficacy. Since the present patient was hoping to have a second child, treatment for osteoporosis was initiated by the use of an active vitamin D preparation, though that showed an insufficient therapeutic effect, which necessitated reconsideration. Because of the high bone affinity and placental passage of BPs [16], as well as the possibility of overshooting without post-treatment for osteoporosis with denosumab, those were not chosen. This was a case of severe osteoporosis with multiple vertebral compression fractures; thus, it was decided to switch to daily teriparatide, which has a short half-life and is the most desirable treatment in terms of efficacy, convenience, and lack of long-term risk for those patients who may benefit from treatment [17].

**Figure 3:**
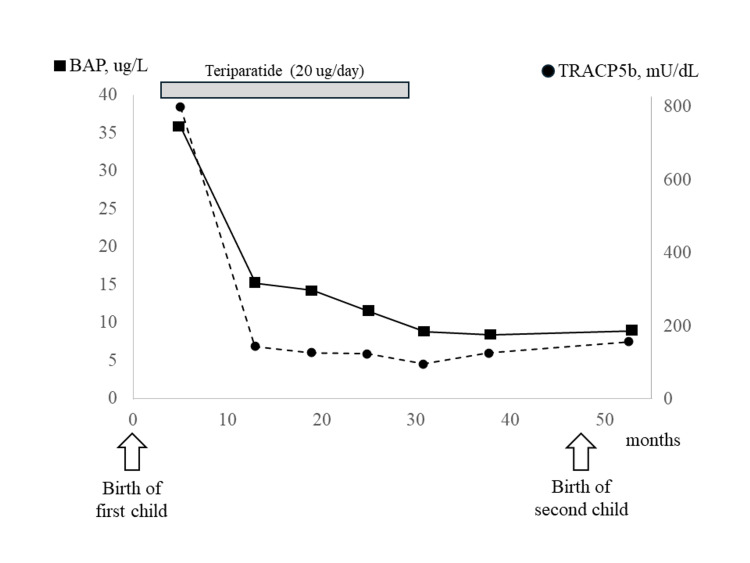
Clinical course of pregnancy and lactation-associated osteoporosis (PLO) treatment and bone metabolic markers. Following treatment intervention with teriparatide, the bone metabolism markers bone-specific alkaline phosphatase (BAP) and tartrate-resistant acid phosphatase (TRACP)5b were markedly decreased, then remained stable without elevation following birth of the second child.

Teriparatide is an osteogenesis-promoting agent, and both bone resorption and bone formation markers usually increase with its use for therapeutic intervention, such as in postmenopausal osteoporosis patients. However, bone metabolism markers, including the bone resorption marker tartrate-resistant acid phosphatase (TRACP)5b and the bone formation marker bone-specific alkaline phosphatase (BAP), did not increase but rather showed a remarkable decrease throughout the treatment period (Figure 3).

Since menstruation had resumed at the time of treatment intervention, it was considered that restoration of ovarian function caused suppression of bone metabolic turnover, which also affected the transition of bone metabolic markers. With teriparatide treatment, lumbar spine BMD (YAM) increased by 22% and femur BMD (YAM) by 15% as compared to their largest decreases. There is no clear evidence regarding the timing of fertility after treatment with teriparatides. Because of concerns about the recurrence of PLO in this case, the patient was advised to resume trying to conceive after maintaining BMD for six months following the end of teriparatide treatment.

Pregnancy experience has been reported to be a moderating factor against the future onset or exacerbation of osteoporosis [18]. On the other hand, a history of PLO has a negative effect on bone prognosis and patient psychology. Kyvernitakis et al. [19] reported that 26 of 107 (24.3%) PLO patients followed for an average of six years developed new fractures, of whom 30 of 81 who received medical treatment became pregnant again, with 13 then choosing abortion and six of 17 who gave birth to another child showing recurrent PLO. Although more than 70% of PLO patients are diagnosed at the time of their first pregnancy [19,20], the low number of reported births after diagnosis and the high rate of elective abortions after another conception suggest that many decide to abandon their next pregnancy and delivery plans after suffering from this difficult condition.

## Conclusions

The development of PLO is a distressing experience for patients and their families and interferes with family planning. In the present case, aggressive therapeutic intervention for osteoporosis in a PLO patient was obtained, who then successfully achieved a new pregnancy. Further research is needed to elucidate the etiology of PLO and establish an effective treatment.
